# The Impacts of Coordinated-Bilateral Ball Skills Intervention on Attention and Concentration, and Cardiorespiratory Fitness among Fourth-Grade Students

**DOI:** 10.3390/ijerph182111634

**Published:** 2021-11-05

**Authors:** Weiyun Chen, Xiaozan Wang, Xiangli Gu, Jun Chen

**Affiliations:** 1 School of Kinesiology, University of Michigan, Ann Arbor, MI 48109, USA; 2 School of Physical Education and Health, East China Normal University, Shanghai 200241, China; xiaozanwang@163.com (X.W.); michaelsara2011@163.com (J.C.); 3 Department of Kinesiology, University of Texas at Arlington, Arlington, TX 76109, USA; xiangli.gu@uta.edu

**Keywords:** coordinated aerobic movements, coordinated-bilateral ball skills, coordinated-bilateral motor skills, cognitive attention, cardiorespiratory fitness

## Abstract

Background: Both cognitive function and cardiorespiratory fitness are significant correlates of physical and mental health. The exploration of innovative school-based PA intervention strategies to improve cognitive function and cardiorespiratory fitness is of great interest for researchers and school educators. This study aimed at examining the effectiveness of the coordinated-bilateral ball skills (CBBS) intervention in improving cognitive function and cardiorespiratory fitness among 4th-grade students. Methods: This study used a two-arm, quasi-experimental research design. The students (*n* = 347) in the intervention group received 16-weeks of CBBS intervention lessons in basketball and soccer. The students (*n* = 348) in the comparison group received 16-weeks of regular basketball and soccer lessons. All participants were pre- and post-tested with the d2 Test of Attention and the Progressive Aerobic Cardiovascular Endurance Run (PACER) test before and after the 16-week CBBS intervention. The data were analyzed by means of descriptive statistics and linear mixed models. Results: The linear mixed models yielded a marginal significant interaction effect of time with the group in their concentration (*F*_(1, 680.130)_ = 3.272, *p* = 0.071) and a significant interaction effect of time with the group in their attention span (*F*_(1, 785.108)_ = 4.836, *p* = 0.028) while controlling for age and the baseline concentration score. The linear mixed model also revealed a significant main effect of time in focused attention (*F*_(1670.605)_ = 550.096, *p* = 0.000), attention accuracy (*F*_(1, 663.124)_ = 61.542, *p* = 0.000), and cardiorespiratory fitness (*F*_(1, 680.336)_ = 28.145, *p* = 0.000), but no significant interaction effect. Conclusions: The CBBS group demonstrated a significant improvement in concentration performance and attention span over time, compared to the comparison group. Both groups improved their focused attention and attention accuracy as well as cardiorespiratory fitness over time. This study suggests that teaching ball skills in team sports for extended periods is instrumental to developing cognitive functions and cardiorespiratory fitness, though the CBBS lessons resulted in greater improvement in concentration performance and attention span.

## 1. Background

Physical activity (PA) is beneficial to improving cognitive function and cardiorespiratory fitness in school-aged children [1,2,3]. Attention, a key indicator of cognitive function, involves processing speed, focused attention, attention accuracy, concentration performance, and sustained attention [4]. Attention is essential to carrying out cognitive processes such as information processing and problem solving [3,4,5]. Therefore, attention is a bedrock to students’ successful academic performance and achievement, and adaptive academic and social behaviors [4,5]. Cardiorespiratory fitness is the efficiency of the circulatory and respiratory systems and the skeletal muscles to deliver and use oxygen needed for energy production during PA [6]. Cardiorespiratory fitness is a key indicator of cardiometabolic health and is strongly associated with cognitive function [7,8]. Both cognitive function and cardiorespiratory fitness are significant correlates of physical and mental health [3,6]. Therefore, an exploration of innovative school-based PA intervention strategies to improve cognitive function and cardiorespiratory fitness is of great interest for researchers and school educators.

Emerging studies have begun using coordinated-bilateral aerobic movements, motor skills, and sport games as PA intervention strategies to promote cognitive function in school-aged children [4,5,6,7,8,9,10,11]. Coordinated-bilateral aerobic movements involve using eye–hand coordination, arm–leg coordination, and spatial orientation to perform body movements with two or more body parts while getting the heart, lungs, and muscles to work [12,13,14]. Coordinated-bilateral motor skills are performed using the coordination of both hands, arms, legs, or feet; coordination of eye–hand and both sides of the body parts; coordination of one side of a body part at a time, and/or switching to the other side to manipulate and move objects (e.g., balls, rackets) by crossing the midline of the body [4,15]. Coordinated aerobic sport games require complex cognitive processes for students to cooperate with teammates, anticipate the movements of teammates and opponents, and use game strategies to apply skills in dynamic and changing game situations [10,11]. Engaging in coordinated-bilateral movements, motor skills, and sport games uses and activates the cerebellum and prefrontal cortex across both hemispheres of the brain [16,17,18].

Studies have examined the effects of coordinated-bilateral movements, motor skills, and sport games interventions in physical education (PE) classes on improving cognitive functions in school-aged children. Budde et al. [15] found a significant acute effect of coordinated-bilateral movement in physical education (PE) lessons on increasing children’s focused attention and attention accuracy assessed with d2 Test of Attention. Schmidt et al. [16] reported that fifth-grade students, who completed the coordinated-movements in a PE lesson showed, 90 min later, a significant increase in their processing speed, attention accuracy, and concentration performance as tested with the d2 Test of Attention, compared to their attention performance tested immediately after the lesson. Harris et al. [4] indicated that fifth grade students significantly increased their processing speed, focused attention, concentration performance, and sustained attention after 4-weeks of, 6 min daily coordinated-bilateral physical activity breaks in their classroom. Chang et al. [9] found that students aged 6–7.5 years, who completed an 8-week coordinative soccer exercises program (two 35-min PE lessons per week), exhibited faster reaction times and more accurate responses in inhibitory tasks compared to their baseline test. Westendorp et al. [11] examined the effects of a 16-week ball skill intervention on motor skill competency and executive functions of 7–11-year-old children with learning disorders. These children showed significant improvement in ball skill competency and problem solving skills, compared to the children in the control group. Pesce et al. [10] designed a 6-month enriched PE intervention using various age-appropriate PA games, motor coordination, and aerobic fitness activities. The children aged 5–10 years in the enriched PE intervention group showed positive changes in ball skills and inhibition control compared to the children in the control group receiving traditional PE programs. These studies indicated that cognitively challenging coordinated movements, motor skills, and sport games are promising intervention strategies for the joint development of motor skills and cognition [9,10,11]. However, none of these studies have examined whether or not the intervention strategies are instrumental to improving cardiorespiratory fitness in school-aged children. 

Studies have shown that children with healthy cardiorespiratory fitness demonstrated higher levels of cognitive function such as processing speed, response accuracy, memory, and problem solving, compared to lower fit children [19,20]. While cardiorespiratory fitness is an essential predictor of cognitive function and physical and mental health during childhood, unfortunately, 60% of youth in the United States have unhealthy cardiorespiratory fitness. Cardiorespiratory fitness has declined over the past six decades [6]. Therefore, an exploration of whether innovative approaches such as coordinated-bilateral motor skill intervention strategies can produce concurrent positive effects on cognitive function and cardiorespiratory fitness among school-aged children is of great importance. 

The purpose of this study is to examine the effectiveness of the coordinated-bilateral ball skills (CBBS) intervention in improving cognitive function and cardiorespiratory fitness among 4th-grade students. We hypothesized that students in the CBBS group would show significant improvement in cognitive function and cardiorespiratory fitness from pre- to post-test compared to the students in the comparison group. 

## 2. Methods

### 2.1. Participants

A total of 743 fourth-grade students (Mean_age_ = 9.62, SD_age_ = 0.642; boys = 415 vs. girls = 325, with 3 missing an identification of gender) from 16 fourth-grade PE classes at five elementary school voluntarily participated in the study. The inclusion criteria for eligible participation included: (1) being fourth-grade students in the class taught by either the intervention PE teacher or the comparison PE teacher, (2) consent/assent to participate in the study, (3) being able to complete all outcome measures without physical, cognitive, and mental constraints. However, those students who did not meet the inclusion criteria from either the intervention or the comparison classes, were allowed to participate in all class activities during the school-scheduled PE lessons. They did not participate in the pre- and the post-test. Trial registration: retrospectively registered, ClinicalTrials.gov Identifier: NCT05033197.

We received approval letters from the five schools’ principals, who agreed with the study protocols and granted permission for their PE teachers and fourth-grade classes of students to participate in the study. Five PE teachers (one per school) consented to serve as intervention teachers because they met the eligible criteria. These criteria included: (1) having a record of attending at least a 3-day national PE teacher workshop for standards-based curriculum and instructions in the past year; and (2) agreeing to attend a 5-day study training course following the terms of the CBBS curriculum, instructions, and assessments. In addition, five PE teachers (one per school) consented to participate in the comparison, non-intervention group. The University Institutional Review Board-Health Sciences and Behavioral Sciences (IRB-HSBS) approved the study protocols (HUM00149529), and we obtained the students’ parental signed consent forms prior to the start of the study. 

### 2.2. Study Design

**Figure 1:**
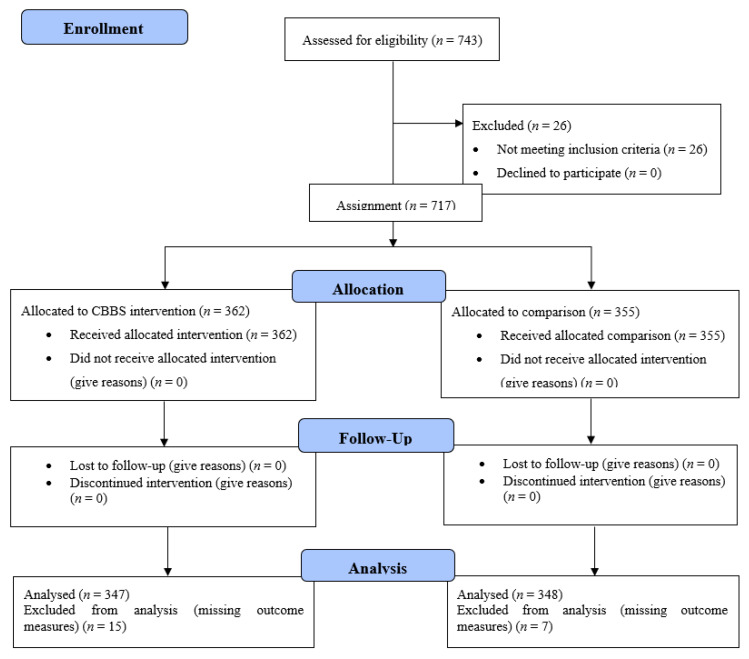
Recruitment and study design diagram.

In this two-arm study, using a quasi-experimental research design, an intact fourth-grade class was used as the cluster unit of analysis nested in the participating school. Students in eight fourth-grade classes (two classes from each of three schools and one class from each of two schools) taught by the five intervention PE teachers were assigned into the CBBS intervention group. On the contrary, students in eight fourth-grade classes (two classes from each of three schools and one class from each of two schools) taught by the five comparison PE teachers were assigned into the comparison group. All participants were pre- and post-tested in the study outcome variables before and after the 16-week CBBS intervention. Figure 1 outlines the participants in each condition over the course of the study using a Consolidated Standards of Reporting Trials (CONSORT) Diagram. 

### 2.3. Sample Size

We calculated the study sample size with an effect size, d = 0.30 for children’s attention and concentration [4], two tails of an alpha level of 0.05, and power of 0.80 using G*Power 3.1.9.7 software. The results revealed that the total required sample size of the study is 352, with 176 students in the intervention group and the same number for the comparison group. Based on our previous study’s adherence rate of greater than 90%, and an estimated program dropout rate of 20%, we need to recruit the targeted sample size of 422 children. 

### 2.4. Teachers Training

To help equip the five PE teachers who agreed to serve as the intervention teachers with the knowledge and skills necessary for implementing the CBBS intervention lessons, we conducted a 5-day (3 h 30 min/day) teacher training session in the study protocols and teaching methods of the CBBS lessons prior to the start of the school year. Details of the training content will be provided upon request.

### 2.5. CBBS Intervention

**Figure 2:**
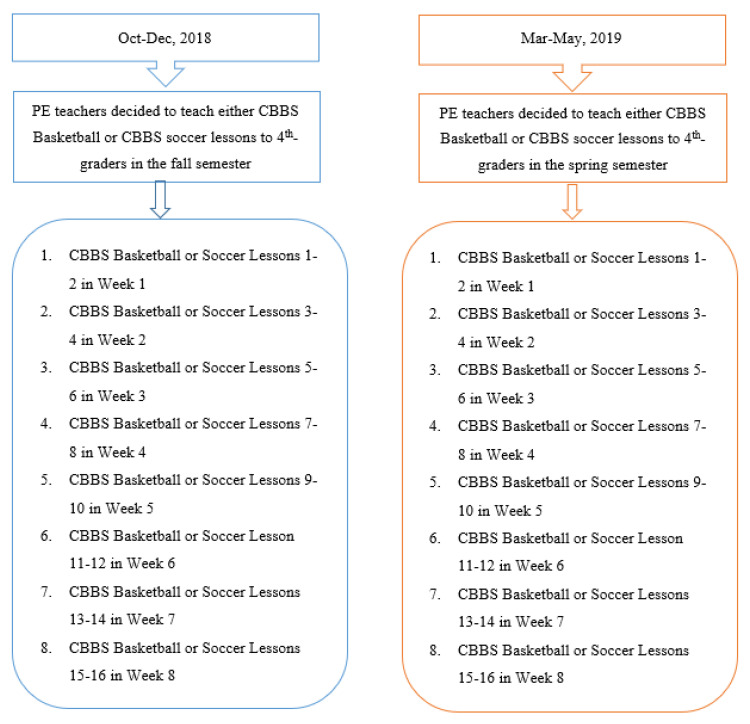
CBBS intervention protocols.

The students in CBBS intervention group received 16-weeks of intervention, including 16 CBBS lessons in the basketball unit (two 40-min lessons/week, 8 weeks) and 16 CBBS lessons in the soccer unit (two 40-min lessons/week, 8 weeks) during the school year (see Figure 2). We designed 32 CBBS lesson plans (16 lesson plans per unit) for the PE teachers to engage students in learning coordinated-bilateral aerobic movements and ball skills in both skill practice and game-like situations within the progressively structured learning environment. Each structured CBBS lesson plan consists of three key components:

(1) Warm-up (7–10 min): focusing on coordinated-bilateral aerobic movements. For example, running forward from the original spot to the next spot, then running for 6 quick steps on the spot, running backward to the original spot, and running for 6 quick steps on the spot again. Repeat this running pattern for 3–5 times.
(2) CBBS skill-building tasks (15–20 min): focusing on the coordinated-bilateral ball skills in combination with locomotor skills. For example, dribbling with the writing hand in place, then changing hand to dribble in place for one minute. Dribbling with writing hand while running on a straight pathway and then changing hand to dribble while running on the straight pathways for two minutes. Dribble with alternating hands while running for two minutes.
(3) Skill-applying tasks (7–10 min): focusing on applying CBBS in cognitively challenging, dynamic, and changing environments. For example, playing a dribble tag game with groups of four students within the designated playing area by walking only, then running.

With the structured lesson plan for each CBBS lesson, the students initially engaged in a variety of coordinated-bilateral aerobic movements (different running patterns), then learned and practiced coordinated-bilateral ball skills in simple traveling situations, and next performed ball skills in more complex and changing game-like situations using both limbs alternatively. While the PE teachers taught the same CBBS curriculum content using the same lesson structure, they had the freedom to modify and adjust specific tasks, the organization of tasks, and the pace of the task progression based on elements such as the class size, available facilities and equipment, and students’ ongoing learning responses. 

### 2.6. Control Condition

The students in the comparison group participated in their regular PE classes. The PE teachers who did not receive the intervention training taught the students 16 basketball lessons and 16 soccer lessons (two 40-min lessons/week, 8 weeks per unit) using their usual methods, within the same timeframe as the intervention group during the school year.

### 2.7. Outcome Measures

The study outcome measures were conducted for both groups at the pre- and the post-test times. In addition, we collected the students’ demographic information including their school, class, age, and sex on the demographic questionnaire at the baseline. 

Progressive Aerobic Cardiovascular Endurance Run (PACER)^®^ test (The Cooper Institute, Dallas, TX, USA). A valid FITNESSGRAM PACER^®^ test (The Cooper Institute, Dallas, TX, USA) (15-m version for school-aged children) was used to assess students’ cardiorespiratory endurance during a PE lesson in the school gymnasium. For this multistage shuttle run test, students ran back and forth across a 15 m distance within their own lane marked by cones before and/or on the sound of beep at a specified pace that increases by the minute for as many laps as they can. Students continue this running pattern until they fail to reach the line before the sound of the beep for the second time. A student’s score on the test is the number of laps completed successfully. The age- and gender-specified cut-off criteria [21] were used to categorize participants into the following two groups: the health fitness zone group (HFZ; PACER lap ≥ 21) and the need-improvement group (NI; PACER lap < 21), respectively. 

D2 Test of Attention. The d2 Test of Attention is a standardized paper and pencil letter-cancellation test that measures the neuropsychology performance of the students in the areas of sustained and selective attention and concentration [22]. It consists of 14 lines of 47 randomly mixed letters “d” or “p” with 1–4 dashes arranged individually or in pairs above or below the character. The d2 Test allows students 20 s per line to identify the letter “d” with two dashes, either above, below or with one dash on the top and one on the bottom. Distractors appear in two forms, as more or less dashes above or below the “d”, and the letter “p” [22]. The students took around 5 min to complete the d2 test.

Four parameters of the d2 Test were used for the data analysis in this study, including (1) TN-E (focused attention): the total number of symbols processed minus the total number of error, (2) CP (concentration performance): the total number of correct responses minus commission errors. It measures the ability to attend to stimuli while disregarding other irrelevant tasks, (3) E% (attention accuracy): the sum of omission and commission errors divided by the total number of items processed, and (4) FR (attention span): which measures the ability to sustain attention to stimuli. It is determined using the line with the highest number of symbols processed minus the line with the lowest number of symbols processed. The d2 Test had high test-retest reliability coefficients for all of the parameters, ranging from 0.95 to 0.98. The test values for criterion, construct, and predictive validity were stable over the course of 23 months after the initial test [23].

### 2.8. Process Evaluation

To allow the PE teachers to implement the CBBS intervention as planned, we conducted a process evaluation of attending the study training and the intervention fidelity. First, we recorded the PE teachers’ attendance of the 5-day teacher training by collecting the sign-up sheet for attendance on each training day. Second, to assess the intervention fidelity, the extent to which the PE teachers taught the CBBS lessons as planned for the 32 CCBS lesson over the 16 weeks, we collected the Weekly Teaching CBBS Lessons Logs completed by each intervention PE teacher at the end of each unit. Third, to assess the implementation fidelity quality and how well the PE teachers taught the CBBS lessons to their students using quality instructional strategies, the PE teachers were asked to use the Assessing Intervention Fidelity Instrument (AIFI) to self-evaluate their teaching for one CBBS lesson every two weeks. The AIFQ was designed for teachers to self-evaluate four indicators of the Fidelity of Implementation: Adherence (three items), Quality of Delivery (six items), Program Specificity (two items), and Students Responsiveness (three items) on a 5-point rating scale (5 = always, 4 = often, 3 = sometimes, 2 = rarely, 1 = never) [24].

### 2.9. Data Analysis

Prior to statistical analysis, we screened the data set to identify the outliers of the d2 test and the PACER test using the SPSS_Explore (Version 27.0; IBM Cooperation, Armonk, NY, USA) and identified missing data on the two tests using the listwise deletion method. The screening resulted in the deletion of 22 cases (15 in the intervention group vs. 7 in the comparison group) and 695 cases in the final data set.

We computed descriptive statistics of each outcome variable (dependent variable) including TN-E, CP, E%, and FR on the d2 Test and PACER test score at the pre-test and post-test stage for each group. Each outcome variable at the two tests met the normality criteria for skewness and kurtosis [25]. An independent sample *t*-test was performed to examine if there were any baseline differences in each outcome variable between the intervention and the comparison groups. To examine the intervention effect on each outcome variable, linear mixed models were conducted for each outcome variable separately while considering students nested in class, nested in school as random effects. The independent variables of each model included the main effect of time (pre-test vs. post-test) and group (intervention vs. comparison), and the interaction effect of time and group. The significant covariates at the baseline between the two groups were controlled for in each model. A statistically significant level for all of the analyses is set at *p* < 0.05. All statistical analyses were performed using IBM SPSS 27 (Version 27.0; IBM Cooperation, Armonk, NY, USA).

To analyze the PE teachers’ attendance rate of the teacher training, we divided the number of days attended by 5 days of the teacher training for each teacher and then computed the average rate. Similarly, to analyze the adherence rate of teaching the CBBS lessons, we divided the number of CBBS lessons taught by 32 CBBS lessons as planned for each teacher and then computed the average rate. To analyze the intervention fidelity quality of teaching the CBBS lessons, we computed the mean score for each sub-scale (indicator) and the total scale of the AIFI among 40 CBBS lessons self-evaluated by the PE teachers.

## 3. Results

### 3.1. Baseline Characteristics and Results of the Process Evaluation 

Table 1 presents the baseline characteristics and outcome variables by group. Regarding the four baseline outcome variables on the d2 Test, independent sample *t*-tests revealed that the experimental group scored significantly higher than the comparison group in TN-E (Focused Attention) and CP (Concentration Performance) (*t* = 2.575, *p* < 0.01; *t* = 2.825, *p* < 0.01). The higher numbers of Focused Attention and Concentration Performance indicate the better performance on the d2 test. In contrast, independent sample *t*-tests showed no significant difference in E% (Attention Accuracy) and FR (Attention Span) between the two groups at the baseline (*t* = −0.990, *p* > 0.05; *t* = 0.173, *p* > 0.05). The lower score represents a better accuracy and attention span. For the baseline PACER test, an independent sample *t*-test revealed no significant difference in the mean score between the two groups (*t* = 1.787, *p* > 0.05). 

All five PE teachers attended each day of the 5-day study training course. Regarding the adherence to teaching the CBBS lessons, the PE teachers self-reported that the average adherence rate was 96% (30.8 out of 32 CBBS lessons). Regarding the intervention fidelity quality of teaching the CBBS lessons, the results showed that the PE teachers almost always demonstrated the four indicators of the intervention fidelity quality, which are Adherence, Quality of Delivery, Program Specificity, and Students Responsiveness across 40 CBBS lessons (Table 2). 

### 3.2. Results of Mixed Linear Models 

**Figure 3:**
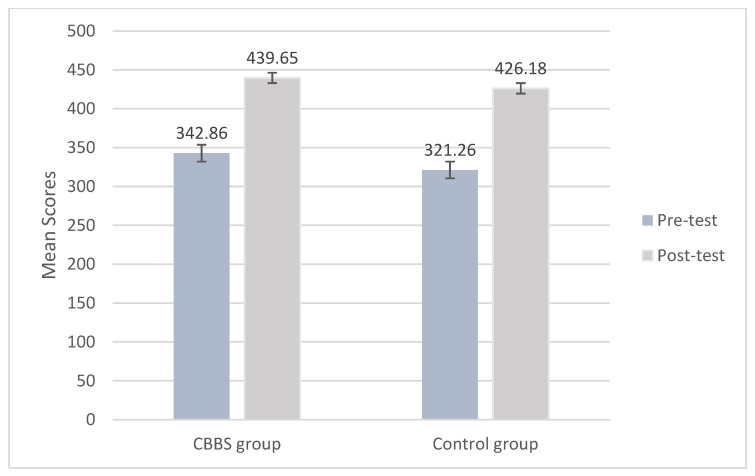
Mean scores of focused attention at pre-test and post-test by group.

**Figure 4:**
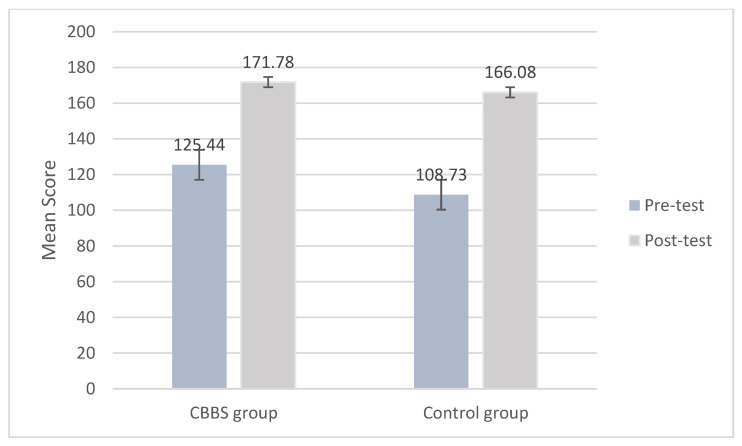
Mean scores of concentration at pre-test and post-test by group.

**Figure 5:**
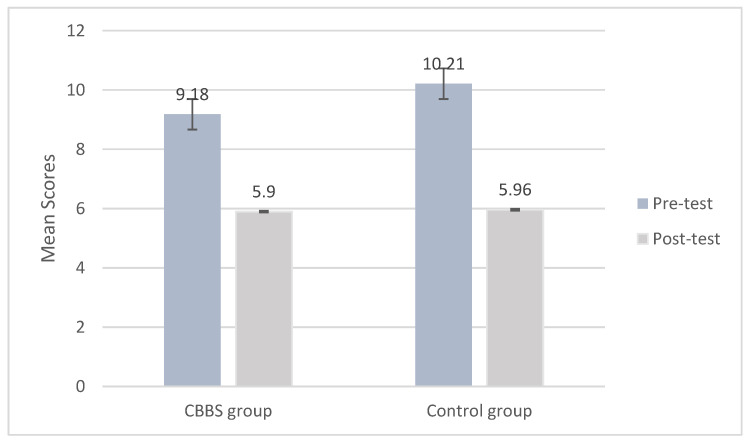
Mean scores of attention accuracy at pre-test and post-test by group.

**Figure 6:**
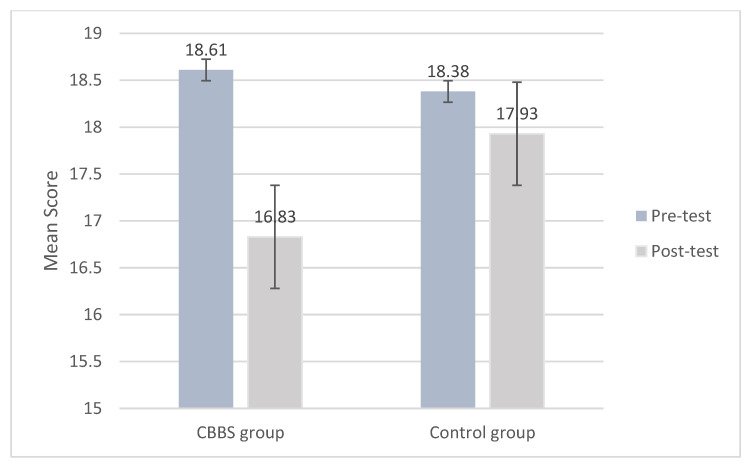
Mean scores of attention span at pre-test and post-test by group.

**Figure 7:**
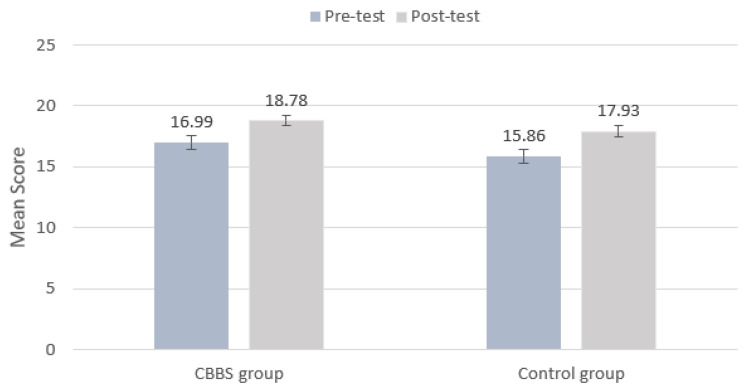
Mean scores of PACER test at the pre-test and post-test by group.

Figures 3–7 present the mean scores of the five outcome variables at the pre-test and the post-test stage between the two groups. Table 3 shows the results of linear mixed models with both a class and a school as random intercept effects. The linear mixed model reveals a significant main effect of time and group in the focused attention (*F*_(1670.605)_ = 550.096, *p* < 0.01; *F*_(1, 679.636)_ = 4.753, *p* < 0.05), but does not reveal a significant interaction effect between time and group (*F*_(1, 670.605)_ = 0.764, *p* > 0.05) while controlling for the following baseline significant measures: age and focused attention. The ICC of the focused attention is 0.38. The results indicate that both groups significantly increased their performance in focused attention over time. Overall, the intervention group had a higher level of focused attention compared to the control group, regardless of time (Figure 3). However, the changes in focused attention between the two groups over time did not reach a significant level (Figure 3). 

Similarly, the linear mixed model yielded a significant main effect of time and group in concentration (*F*_(1, 680.130)_ = 303.936, *p* < 0.01; *F*_(1, 667.253)_ = 4.75, *p* < 0.05), and a marginal significant interaction effect between time and group (*F*_(1, 680.130)_ = 3.272, *p* < 0.10) while controlling for the following baseline significant measures: age and concentration (Table 3). The ICC of the concentration performance is 0.21. The results indicated that although the two groups significantly improved their performance in concentration over time, the intervention group showed more improvement in concentration compared to the control group over time at a marginally significant level (Figure 4). The results indicated a positive intervention effect on concentration. 

The linear mixed model indicated that there was a significant main effect of time in attention accuracy (*F*_(1, 663.124_) = 61.542, *p* < 0.01), but a significant group effect (*F*_(1, 684.402)_ = 0.63, *p* > 0.05) or a significant interaction effect of time with group (*F*_(1, 663.124)_ = 1.021, *p* > 0.05) were not observed (Table 3). The ICC of the attention accuracy is 0.37. The results indicated that the two groups significantly improved their performance in attention accuracy over time. Although, the intervention group demonstrated better performance compared to the control group, however, no significant group difference in attention accuracy was found over time (Figure 5). 

The linear mixed model found no significant main effect of time (*F*_(1, 785.108)_ = 1.971, *p* > 0.05) and group (*F*_(1, 1169.175)_ = 0.718, *p* > 0.05) on the attention span. However, there was a significant interaction effect between time and group (*F*_(1, 785.108)_ = 4.836, *p* < 0.05). The ICC of the attention span is 0.18. The results indicated that the intervention group showed a significant performance improvement in attention span (i.e., better ability to sustain attention to stimuli) over time compared to the control group, showing a positive intervention effect on attention span (Figure 6). 

The Linear Mixed Model revealed a significant main effect of time (*F*_(1, 680.336)_ = 28.145, *p* < 0.01) on cardiorespiratory fitness. The ICC of the cardiorespiratory fitness is 0.49. The results indicated that the two groups significantly increased their performance in cardiorespiratory fitness from baseline to the post-test (Figure 7). There was no significant main effect of the group and interaction effect between time and group (*F*_(1694.605_) = 2.508, *p* > 0.05; *F*_(1680.336)_ = 0.063, *p* > 0.05). The ICC of the cardiorespiratory fitness is 0.49.

## 4. Discussion

This study was central to examining the effectiveness of the 16-week CBBS intervention in improving cognitive function and cardiorespiratory fitness among 4th-grade students, compared to the 4th-grade students attending regular PE classes. As was expected, the students who participated in the CBBS intervention showed noticeable improvements in their concentration and attention span from the baseline to the post-test, compared to the students in the comparison group. Consistent with the previous studies, the results indicate that bilateral coordinated movements and motor skills are beneficial to improving concentration performance and attention span in elementary school students [4,7,26,27,28].

While focusing on helping students learn and practice regular PE contents such as basketball and soccer skills, the intervention PE teachers deliberately encouraged the students to use both sides of the body to engage in a variety of learning tasks during the 32 CBBS intervention lessons. The PE teachers invited the students to initially use their dominant limbs (e.g., writing hand or right foot) to dribble the ball, and then invited them to use their non-dominant limbs to work on the same learning task. The PE teachers used this alternative pattern to engage students in sequential learning tasks during the warm-up and the skill-practice episodes. During the skill-application episode, the students used multiple body parts simultaneously to perform game-like and/or modified game tasks in a dynamic and changing environment in each CBBS intervention lesson. As students dribbled the ball with both hands alternatively in basketball and dribbled the ball with both feet alternatively in soccer, they used both sides of the body and engaged both hemispheres of the brain simultaneously. In reviews of a relationship between motor skills that require coordination, bilateral or cross-lateral motion, and executive functions, Jensen [29,30] indicates that both limbs and both sides of the brain work together to help establish the maturation of movement and cognitive function. The ability to coordinate both hemispheres of the brain helps facilitate other skills such as reading, writing, attention, focus, and memory [29,30]. Budde et al. [15] found that students participating in a single bout of 10-min coordinated bilateral ball skill task significantly increased their performance in focused attention and attention accuracy compared to their counterparts receiving non-coordinated bilateral exercises for the same duration. In other words, children do not often learn well when both sides of the body and both hemispheres of the brain cannot work together collaboratively [31]. Our findings suggest that PE teachers should intentionally encourage students to use both hands and feet in coordinated bilateral and cross-literal ways to work on a variety of learning tasks when teaching any ball skill instructional units to elementary school students. 

Furthermore, it is important to note that the students in the two groups significantly increased their performance in focused attention, concentration, and attention accuracy over the course of participating in the basketball and soccer lessons. Our findings empirically support that teaching ball skills in sports such as basketball and soccer should be core to PE curriculum content for elementary school students, which corresponds with the national content standards for physical education [32]. When learning and practicing ball skills in basketball and soccer, students need to concentrate on controlling the ball using one limb and/or both limbs alternatively depending on situations. Students also need to make spontaneous cognitive decisions on how to use bilateral and/or cross-lateral body movements and ball skills in order to adapt to a variety of situations, especially in game play. Performing such cognitively challenging coordinated bilateral and/or cross-lateral motor tasks requires prefrontal cortex-dependent executive function skills such as attentional control, information processing, strategic planning, goal setting, and cognitive flexibility [12,16,26]. Neuroimaging studies show that when performing novel and complex cognitively challenging motor tasks in unpredictable and changing environments, the cerebellum and basal ganglia (critical for complex and coordinated movements) and the prefrontal cortex (crucial for cognitive functions) become co-activated and the neural network between the cerebellum, basal ganglia, and prefrontal cortex are connected [12,16,30]. Davis et al. [33,34] found that overweight children aged 7–11 years improved their cognitive function processes after participating in 40-min aerobic sport games daily for 12 weeks and 15-weeks, respectively. Similarly, Schmidt et al. [16] indicated that children aged 10–12 years, who participated in a cognitively and physically demanding team games intervention over six weeks, showed more improvement on cognitive flexibility, compared to the group receiving low physical and cognitive demands. This is because that game play requires the application of motor skills in dynamic, unpredictable and novel situations and the constant adaption of motor skills to dynamic and changing environments [11,28,33,34]. Simultaneously, cognitively challenging game tasks demand that a child deliberately attends to changing game situations, swiftly processes received information, flexibly makes decision plans, and efficiently solves problems for achieving the goal-oriented action. Thus, these tasks help children to develop their cognitive functioning skills, which serve as the capstone for children’s adaptive behaviors, social competency, and academic skills [11,28,33,34]. Our findings suggest that when teaching ball skills, PE teachers not only need to engage students in sequential learning tasks using both limbs alternatively, but also need to engage students in cognitively challenging and skill application tasks within the game-like and/or modified game situations. Synchronously, students’ application of ball skills in game-like and/or modified game situations engages them in an aerobic type of exercise, which is conducive to improving cardiorespiratory fitness. 

Contrary to the research hypothesis stating that the CBBS group would show significant improvement in cardiorespiratory fitness over time compared to the comparison group, the students participating in the 16-week programme, with 16 lessons in the basketball unit and 16 lessons in the soccer unit, regardless of instructional conditions, significantly improved their cardiorespiratory fitness over time. The results, on one hand, indicate that engaging students in coordinated-bilateral aerobic movements such as warm-ups, a variety of coordinated-bilateral ball skill learning tasks during a skill-building practice, and using coordinated-bilateral, cross-lateral ball skills to play games in each CBBS lesson over the course of 16 weeks is beneficial to improving cardiorespiratory fitness. Consistent with our results, Harris et al. [4] found that fifth-grade students (*n* = 31), who engaged in 6-min, daily and coordinated-bilateral movements, five days per week over four weeks, showed significant improvement in cardiorespiratory fitness, compared to the control students (*n* = 56). One the other hand, it is noteworthy that students participating in the basketball and soccer lessons over the 16 weeks also improved their cardiorespiratory fitness. Our results provide empirical support for the national content standards for PE [32] by showing that teaching core PE curriculum content, ball skills in basketball and soccer, over extended period is instrumental for school-aged children to increase their cardiorespiratory fitness. This is because basketball and soccer are typical aerobic types of physical activity and typical moderate-to-vigorous intensity activities [35]. In a review of 20 school-based physical activity interventions, Pozuelo-Carrascosa et al. [8] conclude that both moderate and vigorous types of physical activities are conducive to improving cardiorespiratory fitness for school-aged children, with vigorous physical activity having slightly greater effects. Lending support to the national content standards for PE [32], our study suggests that teaching ball skills in team sports should be continually considered as for to the content of PE. 

While the various merits of this study have been discussed above, the study has several limitations. First, the study used a quasi-experimental research design based on the PE teachers’ preference of serving as the instructors for the intervention group or the comparison group. Future study should use a cluster randomized control trial to conduct such an investigation. Second, the study only targeted 4th-grade students as the study participants. To help in better understanding which grade levels could benefit from CBBS intervention effects on cognitive function and cardiorespiratory fitness, future studies may aim to compare the CBBS intervention effects on the outcome variables among different grade levels. Third, the study focused on examining the effects of CBBS in basketball and soccer units on cognitive function and cardiorespiratory fitness. Future studies may extend this study to investigate the effects of CBBS in individual sports (e.g., tennis, pickle ball, badminton) and coordinated-bilateral movements and motor skills (e.g., non-manipulative skills in dance and yoga, and locomotor skills) on the outcome variables. Lastly, the PE teachers in this study used their observations to monitor the students’ intensity levels of physical activity throughout the lesson. It is acknowledged that the failure of objectively monitoring the intensity levels of physical activity during each PE class, due to a large class size, presents one practical limitation in this intervention study. Future studies may use a wearable technology or an interactive health technology system for the entire PE class to objectively monitor students’ real-time intensity levels of task engagement if a school can secure the necessary funding support. 

## 5. Conclusions

The students in the CBBS intervention group significantly outperformed their counterparts in the comparison group for concentration performance and attention span over the course of 16 weeks. However, the students who participated in the ball skills in basketball and soccer units over 16 weeks, regardless of instructional conditions, all positively improvement in terms of their cognitive function (i.e., focused attention, concentration, and attention accuracy) and cardiorespiratory fitness. Furthermore, the CBBS group did not demonstrate a greater level of improvement in cardiorespiratory fitness than the comparison group. This study suggests that teaching ball skills in team sports for extended periods is instrumental to developing cognitive function and cardiorespiratory fitness, though the CBBS lessons resulted in greater improvements in concentration performance and in the children’s attention span.

## Figures and Tables

**Table 1:** Baseline characteristics of participants by group.

**Variables**	Intervention (*n* = 347) Mean (SD)	Control (*n* = 348) Mean (SD)	*p*-Values
Age	9.57 (0.592)	9.67 (0.685)	0.040 *
Boys: number (percentage)	187 (53.9%)	195 (56%)	
Girls: number (percentage)	159 (45.8%)	153 (44%)	
Focused Attention	342.86 (114.24)	321.26 (106.72)	0.010 **
Concentration	125.44 (69.09)	108.73 (85.87)	0.005 **
Attention Accuracy	9.18 (14.05)	10.21 (12.91)	0.322
Attention Span	18.61 (11.44)	18.38 (21.87)	0.863
Cardiorespiratory Fitness	16.99 (8.51)	15.86 (7.98)	0.074

Note: * *p* < 0.05; ** *p* < 0.01.

**Table 2:** Descriptive statistics of each sub-scale and the total scale of the AIFI among 40 CBBS lessons self-evaluated by the PE teachers.

**Indicators**	Min	Max	Mean	SD
Adherence	2.33	5.00	4.26	0.607
Quality of Delivery	2.83	5.00	4.72	0.031
Program Specificity	2.50	5.00	4.41	0.542
Students Responsiveness	3.00	5.00	4.62	0.469
Total Scale	3.64	5.00	4.51	0.339

**Table 3:** Results of Linear Mixed Models for outcome variables.

**Dependent Variables**	Time		Group		Time * Group	
	*F*	*p*	*F*	*p*	*F*	*p*
Focused Attention	550.096	0.000 **	4.753	0.030 *	0.764	0.383
Concentration	303.936	0.000 **	4.389	0.037 *	3.272	0.071
Attention Accuracy	61.542	0.000 **	0.630	0.428	1.021	0.313
Attention Span	1.971	0.161	0.718	0.397	4.846	0.028 *
Cardiorespiratory Fitness	28.145	0.000 **	2.508	0.114	0.063	0.802

Note: * *p* < 0.05; ** *p* < 0.01

## Data Availability

The data presented this study are available on request from the corresponding author [WC].
